# DPHC From *Alpinia officinarum* Ameliorates Oxidative Stress and Insulin Resistance *via* Activation of Nrf2/ARE Pathway in db/db Mice and High Glucose-Treated HepG2 Cells

**DOI:** 10.3389/fphar.2021.792977

**Published:** 2022-01-17

**Authors:** Xuguang Zhang, Yuxin Zhang, Mingyan Zhou, Yiqiang Xie, Xiujuan Dong, Feihu Bai, Junqing Zhang

**Affiliations:** ^1^ Key Laboratory of Tropical Translational Medicine of Ministry of Education, Hainan Provincial Key Laboratory for Research and Development of Tropical Herbs, Haikou Key Laboratory of Li Nationality Medicine, School of Pharmacy, Hainan Medical University, Haikou, China; ^2^ Traditional Chinese Medicine (TCM) College, Hainan Medical University, Haikou, China; ^3^ The Gastroenterology Clinical Medical Center of Hainan Province, Department of Gastroenterology, The Second Affiliated Hospital of Hainan Medical University, Haikou, China

**Keywords:** DPHC, *Alpinia officinarum*, T2DM, oxidative stress, insulin resistance, Nrf2/are

## Abstract

(R)-5-hydroxy-1,7-diphenyl-3-heptanone (DPHC) from the natural plant *Alpinia officinarum* has been reported to have antioxidation and antidiabetic effects. In this study, the therapeutic effect and molecular mechanism of DPHC on type 2 diabetes mellitus (T2DM) were investigated based on the regulation of oxidative stress and insulin resistance (IR) *in vivo* and *in vitro*. *In vivo*, the fasting blood glucose (FBG) level of db/db mice was significantly reduced with improved glucose tolerance and insulin sensitivity after 8 weeks of treatment with DPHC. *In vitro*, DPHC ameliorated IR because of its increasing glucose consumption and glucose uptake of IR-HepG2 cells induced by high glucose. In addition, *in vitro* and *in vivo* experiments showed that DPHC could regulate the antioxidant enzyme levels including superoxide dismutase (SOD), catalase (CAT), and glutathione peroxidase (GSH-Px), thereby reducing the occurrence of oxidative stress and improving insulin resistance. Western blotting and polymerase chain reaction results showed that DPHC could promote the expressions of nuclear factor erythroid 2-related factor 2 (Nrf2), the heme oxygenase-1 (HO-1), protein kinase B (AKT), and glucose transporter type 4 (GLUT4), and reduced the phosphorylation levels of c-Jun N-terminal kinase (JNK) and insulin receptor substrate-1 (IRS-1) on Ser307 both *in vivo* and *in vitro*. These findings verified that DPHC has the potential to relieve oxidative stress and IR to cure T2DM by activating Nrf2/ARE signaling pathway in db/db mice and IR-HepG2 cells.

## Introduction

Type 2 diabetes mellitus (T2DM) has become a major health burden for humans and is considered to be one of the fifth leading causes of death all over the world (Yu et al., 2015). Almost 80% of patients with T2DM are in developing countries, and the prevalence of diabetes in China accounts for nearly 11%, ranking first in the world (Cheung et al., 2018). In fact, T2DM is mainly associated with insulin resistance (IR) and decreased insulin sensitivity due to impaired insulin signal transduction. IR can lead to a variety of diabetic complications such as diabetic liver injury and diabetic nephropathy (Kim et al., 2009), and these complications would cause severe harm to diabetic patients in the development of the disease. Therefore, safe and effective drug discovery for preventing and reducing IR is of great significance. Relevant evidence showed that a series of signal pathways related to oxidative stress are activated, which exacerbated IR and impaired insulin secretion when blood glucose remains at high levels for a long time (Bhakkiyalakshmi et al., 2015). Oxidative stress is thought to be able to increase the occurrence and development of liver disease in diabetic patients (Kanikarla-Marie and Jain, 2015), and the research clarified that there are numerous oxidative stress markers in the serum of patients with T2DM (Maiese, 2015). Reactive oxygen species (ROS), an important molecule inducing oxidative stress in organisms, has been shown to cause IR by activating the c-Jun N-terminal kinase (JNK) signaling pathway (Yang et al., 2014). The activation of JNK would further affect the phosphorylation of insulin receptor substrate 1 (IRS-1) on Ser307 and block insulin signaling transduction, thereby inhibiting the involvement of GLUT4 in transport and then leading to IR. Malondialdehyde (MDA) is an important oxidative stress marker that reflects body’s antioxidation potential (EL-Beltagi and Mohamed, 2013), and glutathione peroxidase (GSH-Px), superoxide dismutase (SOD), and catalase (CAT) are the main antioxidant enzymes that protect body tissues from harmful ROS (Shen and Kong, 2009; Ighodaro and Akinloye, 2019). In addition, nuclear factor-erythroid 2-related factor 2 (Nrf2) binding to the antioxidant response element (ARE) exerts a key role in the antioxidant response and glucose metabolism. During a short period of oxidative stress, Nrf2/ARE activates phase II detoxification enzymes and antioxidant proteins (Tan et al., 2011) such as SOD, heme oxygenase-1 (HO-1), and NADPH quinone oxidoreductase (NQO1) (Wang et al., 2017) to be taken for a key factor to alleviate oxidative stress and IR (Yang et al., 2014). Hence, Nrf2 activation contributes the inhibition of the complications of diabetes (Uruno et al., 2015). Simultaneously, GSH-Px is also a key target reflecting the degree of activation of Nrf2/ARE (Shen and Kong, 2009). Therefore, overexpression of oxidative-stress-related signaling pathways in T2DM is one of the keys leading to IR and impaired glucose tolerance (Tangvarasittichai, 2015), and activation of Nrf2/ARE signaling pathway could improve IR caused by oxidative stress and consequently treat T2DM.

*Alpinia officinarum* Hance of Zingiberaceae plant is an important edible medicinal herb widely cultivated in the tropical and subtropical areas of Asia (Zhou et al., 2018). Modern pharmacological studies showed that *A. officinarum* had antioxidant, antidiabetic, antiulcer, and other pharmacological effects (Mayachiew and Devahastin, 2008; Lee et al., 2009; Xie et al., 2013). Our original research preliminarily confirmed that *A. officinarum* could significantly reduce random blood glucose levels of T2DM mice induced by high-fat diet combined with STZ-nicotine and improve glucose tolerance (Cheng et al., 2017). Phytochemical research evidence manifests that diarylheptanoids, a class of characteristic components bearing 1,7-diphenylheptane skeleton (Lv and She, 2010), are the main active ingredients in the rhizome of *A. officinarum* and have many biological activities (Ly et al., 2003; An et al., 2008; Zhang et al., 2010; Honmore et al., 2016). Furthermore, (R)-5-hydroxy-1,7-diphenyl-3-heptanone (DPHC) is a diarylheptanoid component with high content in *A. officinarum.* In our pre-experiment, DPHC also significantly reduced blood glucose in T2DM mice, and interestingly, it also improved oxidative stress states of mice (data not shown). However, the underlying molecular mechanism of DPHC exert hypoglycemic action remains to be unknown.

Therefore, we further investigated the hypoglycemic activity and the molecular mechanism of DPHC by improving oxidative stress and IR based on Nrf2/HO-1/JNK/IRS1/PI3K/AKT/GLUT4 signaling pathway. In this study, db/db mice and high glucose-induced IR-HepG2 cells were administered with DPHC to observe its effect on glucose metabolism *in vivo* and *in vitro*. In addition, the possible mechanism of insulin signal transduction pathway was elucidated, which provided experimental basis for further research on the clinical application of DPHC.

## Materials and Methods

### Drug Extraction and Isolation

**FIGURE 1:**
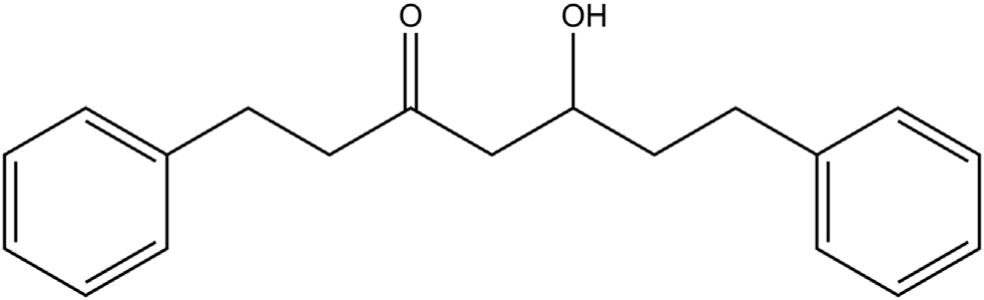
The chemical structure of DPHC.

*Alpinia officinarum* powders (42 kg) were extracted with 10-fold 85% ethanol by heating reflux for three times for 2 h. The solvent was combined and condensed in vacuum to yield a crude extract. After suspension in anhydrous ethanol (10 L), an appropriate amount of crude extract was extracted with ethyl acetate (EtOAc) to obtain the EtOAc extract, and then, it was subjected to silica gel CC eluted with petroleum ether/EtOAc (30:1→10:1, v/v). DPHC was obtained when the column was eluted in the ratio 15:1, and the structure was identified as (R)-5-hydroxy-1,7-diphenyl-3-heptanone based on spectroscopic analysis (^1^H NMR, ^13^C NMR) as originally reported (Cheng, 2016). The purity of DPHC was determined to be over 98% by high-performance liquid chromatography (HPLC) detection. The chemical structure is presented in Figure 1.

### Animals Experiments

At 7 weeks of age, male C57BL/KsJ db/db and db/m mice (Jiangsu Jicui Yaokang Biotechnology Co., Ltd., China) were kept in a room with a 12-h light–dark cycle at 25°C. During the entire experiment, the mice were given free access to food and water in the Laboratory Animal Center of Hainan Medical University. All experimental procedures were implemented under the approval of the Experimental Animal Ethics Committee of Hainan Medical University (No. HYLL-2021-377) and carried out in accordance with the Animal Ethics Regulations of the People’s Republic of China and the Animal Ethics Guidelines of the People’s Republic of China. After a week of adaptation period, nine male db/m mice were assigned to db/m group as a blank control (n = 9), and 36 db/db mice were randomly divided into four groups (n = 9) given different treatments. First, db/db mice in db/db group were applied as a model without any treatment, and db/db mice in the rosiglitazone (RSG) group was medicated with RSG with a dose of 10 mg/kg body weight as a positive control (Sharma et al., 2015). Meanwhile, db/db mice in the two DPHC groups were given DPHC solution with doses of 80 and 140 mg/kg once a day. All mice were given gavage administration in a volume of 0.2 ml/10 g. Moreover, drugs were dissolved in 1% sodium carboxymethyl cellulose solution, and mice in the normal group and model group were given the same amount of solution as the drug groups. The feed were provided by Guangdong Medical Laboratory Animal Center.

Mouse body weight and fasting blood glucose (FBG, fasting for 8 h) were monitored by a calibrated weighing scale and a glucometer (Accu-Chek; Roche Diagnostics, Mannheim, Germany), and blood was collected by caudal vein acupuncture. In addition, dosing was implemented at 4 p.m. every day, and the entire experiment lasted for 8 weeks. At the end of the experiment, the whole blood was collected from the orbital sinus, and serum was separated by centrifugation (3,000 rpm for 15 min) and stored at −20°C. Whereafter, the mice were sacrificed by cervical dislocation, and the liver tissues were collected. Two parts of the left and right lobes of the liver were taken for Western blotting (WB) and PCR analysis, and a small piece of intact liver tissue was taken for pathological section analysis. Finally, all liver tissues were frozen in a −80°C refrigerator for further research.

### Oral Glucose Tolerance Test and Insulin Tolerance Test

After 5 weeks of treatment, oral glucose tolerance test (OGTT) was used to measure blood glucose levels of mice in each group at 0, 30, 60, 90, and 120 min given 2 g/kg of glucose after fasting for 12 h in each group. After 6 weeks of treatment, insulin tolerance test (ITT) was utilized to measure blood glucose levels of mice in each group at 0, 30, 60, 90, and 120 min *via* intraperitoneal injection of insulin with the dose of 1 U/kg after 6 h of fasting. Finally, glucose content was calculated as the area under the curve (AUC) using GraphPad prism 8 (GraphPad Software Inc., San Diego, CA, United States).

### Serum Insulin Level and Biochemical Measurement of Liver Tissue in Mice

Serum insulin was measured using the enzyme-linked immunosorbent assay (ELISA) kit (Cat. number ab277390, Abcam, United Kingdom) according to the instruction provided by the manufacturer. The homeostasis model assessment of IR (HOMA-IR) and insulin sensitive index (ISI) were calculated according to the following formula: HOMA-IR = (fasting serum glucose × fasting serum insulin)/22.5; ISI = ln (l/fasting serum glucose × fasting serum insulin).

A series of biochemical indicators related to oxidative stress in the liver tissues of mice, such as CAT, MDA (Cat. numbers A007-1-1 and A003-1, Nanjing Jiancheng Bioengineering Institute, China), SOD, and GSH-Px (Cat. numbers S0101S and S0065, Beyotime, Institute of Biotechnology, Haimen, China) were tested using the corresponding kit according to the manufacturer’s instructions, respectively.

### Liver Histology and Liver Glycogen Content

The mouse liver tissues used for pathological section analysis were taken out and fixed in 4% formalin at 25°C. After embedding in paraffin, sections (4 µm) were stained with hematoxylin–eosin (HE) for liver pathology evaluation. The level of hepatic glycogen was measured using the Liver/Muscle glycogen assay kit (Cat. number A043-1-1, Nanjing Jiancheng Bioengineering Institute, China) according to the manufacturer’s instruction.

### Cell Culture and Treatment

HepG2 cells (Zhong Qiao Xin Zhou Biotechnology, Co., Ltd., Shanghai, China) were cultured with Dulbecco’s modified Eagle’s medium (DMEM) supplemented with 10% fetal bovine serum (FBS) and 100 U/ml penicillin/streptomycin (Invitrogen, Carlsbad, CA, United States) mixed solution at 37°C and 5% CO_2_. The IR model was induced by high glucose in HepG2 cells as formerly depicted (Ding et al., 2019). Briefly, HepG2 cells were seeded into 96-well plates at a density of 1 × 10^5^ cells∙ml^−1^ and incubated in DMEM with 5.5 mM regular glucose for 24 h. IR model was established by adding 30 mM glucose for an additional 12 h. Subsequently, HepG2 cells were grouped for the *in vitro* experiments, with treating for regular glucose (5.5 mM) as a normal control, treating for high glucose (30 mM) as a model control, and treating for RSG (50 µM) as a positive control, respectively. In addition, DPHC groups were set to three concentrations: 10, 20, and 50 μmol/L. In WB and PCR experiments of Nrf2 and HO-1 proteins or mRNA, ML385 (5 µm), an Nrf2 inhibitor, was added into the DPHC (50 µM) as an additional experimental group.

### Determination of Cell Viability

Cell viability was determined by cell counting kit-8 (CCK-8) (Cat. number C0038, Beyotime, Haimen, China). HepG2 cells were seeded in 96-well plates at a density of 1 × 10^5^ cells∙ml^−1^. After culturing for 24 h, the cells were combined with different concentrations of DPHC for 12 h. A Molecular Devices Spectra Max Plus automatic plate reader (Molecular Device, Sunnyvale, CA, United States) was used to detect the absorbance at 450 nm, and the cell viability was expressed as the ratio of the absorbance of the treated group to that of the normal control group. The optimal concentration of DPHC was selected as the experimental concentration according to the result of this study.

### Glucose Consumption and Glucose Uptake Analysis

HepG2 cells were seeded in 96-well plates and treated with different administration methods. The glucose concentration in culture medium was determined by the glucose oxidase peroxidase method kit (Cat. number F006-1-1, Nanjing Jiancheng Bioengineering Institute, China). The results were calculated as follows: glucose consumption = glucose concentration in blank group (no cells, only medium) − glucose concentration in each group. Lastly, CCK-8 was added to each hole for 1 h, and OD value was measured to correct the number of cells.

Cellular glucose uptake was assessed using the fluorescent glucose analogue: 2-(N-(7-nitrobenz-2-oxa-1,3-diazol-4-yl) amino)-2-deoxy-d-glucose (2-NBDG), as described by Zou et al. (2005) with slight modifications. In short, the cells were seeded in a six-well plate at a density of 2 × 10^5^ cells∙ml^−1^. After administration treatment, 2-NBDG (Invitrogen Life Technologies, Carlsbad, CA, United States) was added at a final concentration of 25 μM at 37°C for 20 min. Then, cells were washed twice with phosphate-buffered saline (PBS) and then immediately detected the fluorescence intensity by flow cytometry (FACS Calibur, Agilent NovoCyte Penteon, United States) after suspending with PBS. Finally, a reasonable estimate of the total glucose uptake was obtained by quantifying the fluorescence.

### Determination of Intracellular ROS, MDA, and SOD Activities

Intracellular ROS level was determined using a kit according to the manufacturer’s instruction (Cat. number S0033S, Beyotime, Haimen, China). HepG2 cells treated in each group were washed three times by PBS, and then, 2′,7′-dichlorodihydrofluorescein diacetate (DCFH-DA) probe was added with a final concentration of 10 µM to each well for 30 min incubation at 37°C. The cells were then washed three times with PBS to remove DCFH-DA that did not enter the cells and immediately measured by flow cytometry. The ROS level was estimated according to the fluorescence.

SOD and MDA levels were measured using the kits (Cat. numbers 121120210106 and 010620200501, Beyotime, Haimen, China). HepG2 cells grown on a six-well plate were collected and lysed with cell lysis buffer on ice. The total protein concentration in the supernatant was determined with BCA protein assay kit (Cat. number P0012, Beyotime, Haimen, China). The relative activities of SOD and MDA were expressed as the ratio of total protein.

### Western Blotting Analysis

Total proteins from HepG2 cells and mice liver tissues were extracted by radioimmunoprecipitation assay (RIPA) lysis buffer (Cat. number P0013C, Beyotime, Haimen, China) containing phosphatase inhibitor (Cat. number P1081, Beyotime, Haimen, China) and protease inhibitor (PMSF) (Cat. number L1913093, Aladdin, Shanghai, China) and then centrifuged at 13,000 rpm for 15 min at 4°C. Protein samples were mixed with 5× loading buffer and denatured at 100°C for 5 min. Proteins were separated by 12% sodium dodecyl sulfate-polyacrylamide and then transferred to polyvinylidene difluoride membranes. The membranes were blocked with 5% nonfat dry milk for 1 h and incubated with different primary antibodies: PI3K (1:1,000), p-PI3K (Tyr458/Tyr199) (1:1,000), AKT (1:1,000), *p*-AKT (Ser473) (1:2,000) (Cell Signaling Technology, Beverly, MA, United States), *p*-Nrf2 (Ser40) (1:2,000), p-IRS1 (Ser307), *p*-JNK (Thr183/Tyr185) (1:1,000) (Affinity, Cambridge, United Kingdom), Nrf2 (1:1,000), HO-1 (1:3,000), JNK (1:2,000), IRS1 (1:1,000), and GLUT4 (1:2,000) (Proteintech, Chicago, IL, United States) at 4°C overnight. After washing with Tris-buffered saline with Tween 20 (TBST), the membrane was incubated with the specific secondary antibody for 1 h at room temperature. Finally, the amounts of the proteins were detected using the ECL chemiluminescence kit (Labgic Technology Co., Ltd., Beijing, China) and Image Lab analysis software (Bio-Rad Laboratories, Inc., Hercules, CA, United States). The protein concentration was determined by BCA protein assay kit. Glyceraldehyde 3-phosphate dehydrogenase (GAPDH), β-actin, and tubulin were used as loading controls.

### Quantitative Polymerase Chain Reaction Analysis

The gene expression levels of *Nrf2*, *GPX1*, *HO-1*, *JNK*, *IRS1*, and *AKT* were detected by quantitative polymerase chain reaction (qPCR). Total RNA was extracted from the liver tissues of mice and HepG2 cells by Eastep^®^ Super Total RNA Extraction Kit (Cat. number LS1040, Shanghai Promage Biological Products Co., Ltd., China), and the quality of extracted RNA was confirmed using a NanoPhotometer-N50 ultra-microspectrophotometer (Implen, Germany). The PCR amplification was performed under the following conditions: the initial activation of the hot-start DNA polymerase at 95°C for 5 min, followed by 40 cycles of the second step (i.e., 95°C for 10 s, 58°C for 20 s, 72°C for 20 s). This is followed by the melting curve stage, and finally, the amplification primers were obtained. The relative expression of each gene was analyzed by the 2^−ΔΔCt^ method, and *GAPDH* was used as the internal reference. The qPCR primer sequences are shown in Table 1.

**TABLE 1:** The qPCR primer sequences.

Primer	Primer sequence (5′ to 3′)	Primer sequence (5′ to 3′)
*Nrf2*-F	**M**-CAGCCATGACTGATTTAAGCAG	**H**-CCAGCACATCCAGTCAGAAACCAG
*Nrf2*-R	**M**-CAGCTGCTTGTTTTCGGTATTA	**H**-AGCCGAAGAAACCTCATTGTCATCTAC
*HO-1*-F	**M**-TCCTTGTACCATATCTACACGG	**H**-CCTCCCTGTACCACATCTATGT
*HO-1*-R	**M**-GAGACGCTTTACATAGTGCTGT	**H**-GCTCTTCTGGGAAGTAGACAG
*GPX1*-F	**M**-GTTTGAGAAGTGCGAAGTGAAT	**H**-CCCTCTGAGGCACCACGGT
*GPX1*-R	**M**-CGGAGACCAAATGATGTACTTG	**H**-TAAGCGCGGTGGCGTCGT
*JNK*-F	**M**-TTGAAAACAGGCCTAAATACGC	**H**-CCAGGACTGCAGGAACGAGT
*JNK*-R	**M**-GTTTGTTATGCTCTGAGTCAGC	**H**-CCACGTTTTCCTTGTAGCCC
*IRS1*-F	**M**-GAGTTGAGTTGGGCAGAATAGG	**H**-CAGCTCACCTTCTGTCAGG
*IRS1*-R	**M**-CCTATCTGCATGGTCATGTAGT	**H**-AGGTCCATCTTCATGTACTCC
*AKT*-F	**M**-TGCACAAACGAGGGGAATATAT	**H**-TGACCATGAACGAGTTTGAGTA
*AKT*-R	**M**-CGTTCCTTGTAGCCAATAAAGG	**H**-GAGGATCTTCATGGCGTAGTAG

### Statistical Analysis

All data were analyzed by GraphPad Prism 8 and SPSS 19.0 software and expressed as means ± standard deviation (SD). One-way ANOVA was utilized to compare differences among multiple groups, and the non-paired *t*-test was used to analyze two groups after homogeneity of variance testing. *p* < 0.05 denoted statistical significance.

## Results

### *In Vivo* Study

#### Effects of DPHC on Body Weight and FBG in db/db Mice During Treatment

**FIGURE 2:**
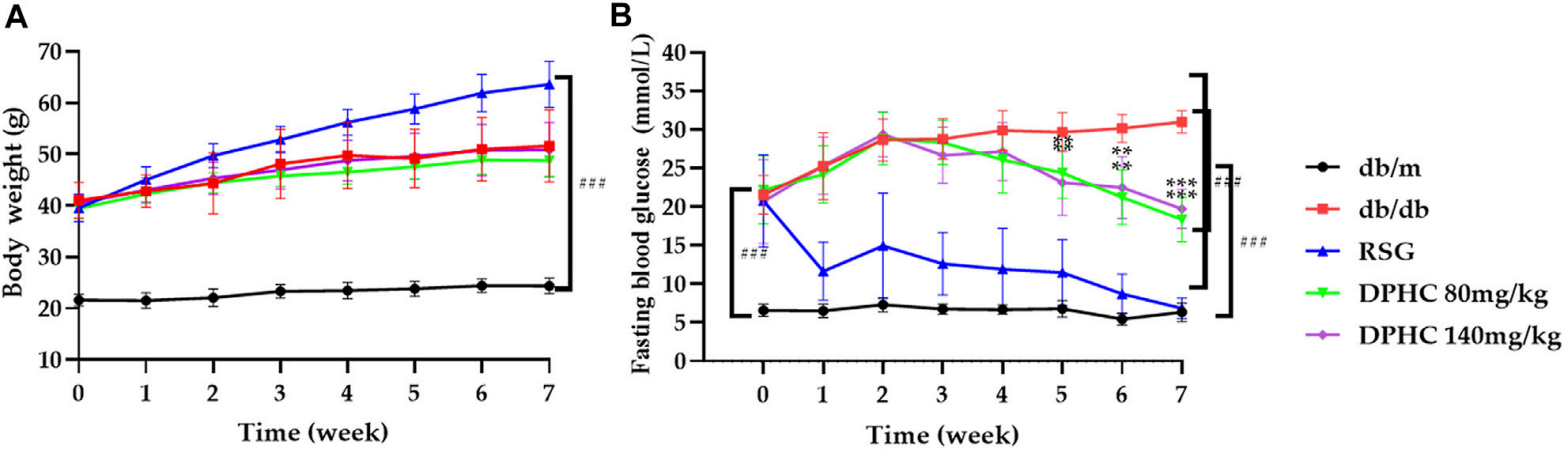
Effects of DPHC on body weight and FBG. **(A)** Body weight. **(B)** FBG levels. All values are expressed as means ± SD, n = 9. **p < 0.01, ***p < 0.001 compared with the db/db group, and ^###^ *p* < 0.001 compared with the db/m group.

As shown in Figure 2A, there was no significant change in body weight of db/m mice, while the weight of other groups were significantly higher than the db/m group (*p* < 0.001) during the experiment. Besides, there was no significant difference between the db/db group and the DPHC groups when the RSG group gained the most weight, which confirmed the side effect of RSG in the treatment for T2DM (Liu et al., 2017). Therefore, these results showed that DPHC had no influence on the body weight of mice.

The changes in FBG in each group are shown in Figure 2B. The results showed that there was no significant difference among the db/db, RSG, and DPHC groups at the beginning of the experiment. In the third week of dosing, the FBG levels of mice in the DPHC (80 and 140 mg/kg) groups started decreasing. At the fifth week of dosing, the FBG levels of the DPHC groups were significantly reduced from that of the db/db group (*p* < 0.01). After that, FBG level in the db/db group increased persistently, while the FBG levels of the DPHC groups continued to decrease with a very significant difference in the seventh week compared with the db/db group (*p* < 0.001). Therefore, DPHC could reduce FBG of db/db mice.

#### Effect of DPHC on OGTT and ITT in db/db Mice

**FIGURE 3:**
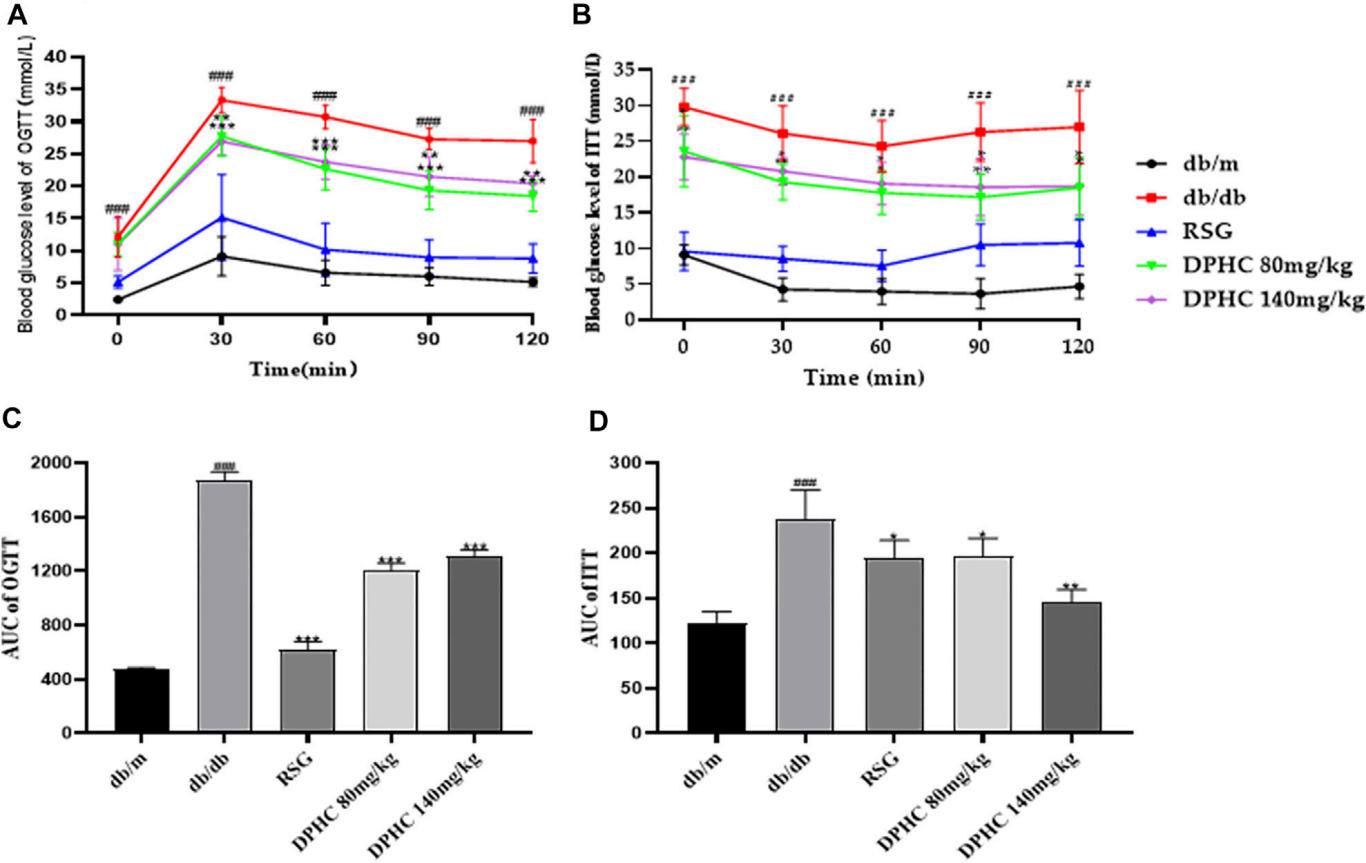
Effects of DPHC on OGTT and ITT in db/db mice. **(A)** The change in glucose level in the OGTT. **(B)** The change in glucose level in the ITT. **(C)** The AUC of the OGTT. **(D)** The AUC of the ITT. The baseline correction of the AUC of OGTT and ITT was conducted based on the lowest blood glucose value of each group. All values are expressed as means ± SD, n = 9. *p < 0.05, **p < 0.01, ***p < 0.001 compared with the db/db group, and ^###^ *p* < 0.001 compared with the db/m group.

The OGTT was used to assess glucose tolerance (Xiao et al., 2011). As shown in Figure 3A, the blood glucose levels of mice in all groups reached the highest value within 30 min after taking glucose orally and then gradually returned to normal within 120 min. The AUC of the db/db group was significantly higher than that of the db/m group (*p* < 0.001) based on the calculation. However, the AUC of the DPHC (80 and 140 mg/kg) groups was significantly lower than that of the db/db group (*p* < 0.05) shown in Figure 3C.

The ITT was utilized to assess glucose clearance (Zou et al., 2005). As shown in Figure 3B, the blood glucose levels of mice in all groups showed a tendency to first drop rapidly and then slowly rose to a normal state after intraperitoneal injection of insulin. The AUC of the db/db group was significantly higher than that of the db/m group (*p* < 0.001). Nevertheless, the AUCs of the DPHC groups were significantly lower than that of the db/db group (*p* < 0.05) shown in Figure 3D. The above results showed that DPHC could reduce the blood glucose levels of db/db mice and improve their glucose tolerance and insulin sensitivity.

In OGTT and ITT, the lowest point of blood glucose curve in each group was used as the baseline value for correction shown in Figures 3C,D.

#### Effect of DPHC on Serum Insulin Level and Oxidative Stress of Liver Tissue in db/db Mice

**FIGURE 4:**
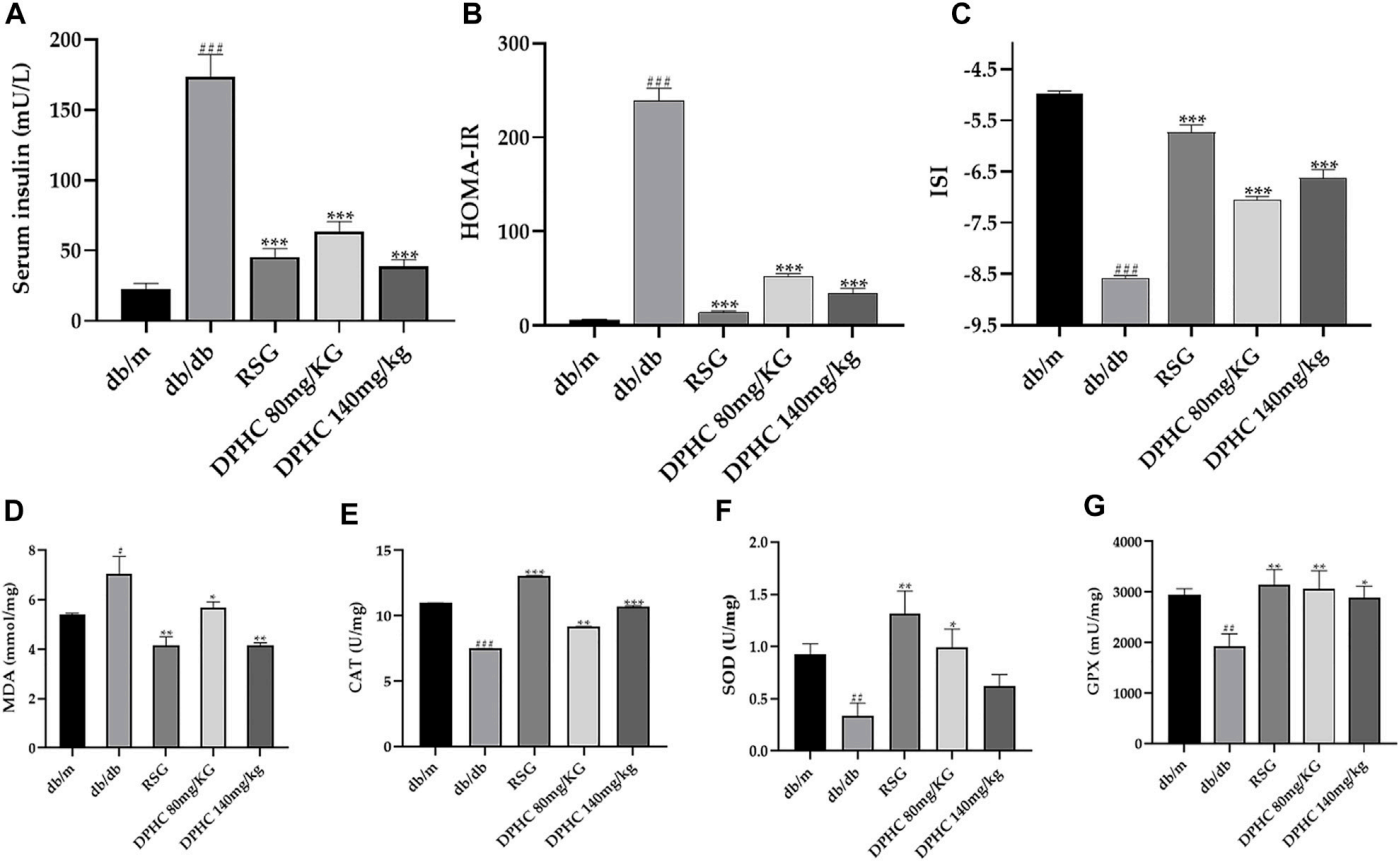
Effect of DPHC on serum insulin level and antioxidant enzymes of liver tissues in db/db mice. **(A)** Serum insulin. **(B)** HOMA-IR. **(C)** ISI. **(D)** MDA. **(E)** CAT. **(F)** SOD. **(G)** GSH-Px. All values are expressed as means ± SD, n = 9. *p < 0.05, **p < 0.01, ***p < 0.001 compared with the db/db group, and ^##^ *p* < 0.01, ^###^ *p* < 0.001 compared with the db/m group.

As shown in Figure 4A, serum insulin concentration of the db/db group was observably increased (*p* < 0.001) compared with the db/m group. Nevertheless, the serum insulin of DPHC (80 and 140 mg/kg) and RSG groups were reduced by 63.6% (*p* < 0.001), 78.0% (*p* < 0.001), and 73.9% (*p* < 0.001), respectively after 8 weeks of administration compared with the db/db group. In addition, the HOMA-IR and ISI were also utilized to explore the improvement effect of DPHC on IR. As depicted in Figures 4B,C, HOMA-IR and ISI of DPHC and RSG groups were significantly decreased and increased (*p* < 0.001), respectively, compared with the db/db group. The aforementioned results indicated that DPHC could improve IR in db/db mice.

Antioxidant ability was evaluated by detecting the changes in antioxidant enzyme activities such as MDA, SOD, CAT, and GSH-Px. As shown in Figures 4D–G, the levels of SOD, CAT, and GSH-Px in the db/db group were markedly reduced (*p* < 0.01), and the level of MDA was significantly increased (*p* < 0.05) compared with the db/m group. Nevertheless, the SOD, GSH-Px, and CAT levels were significantly increased, and the MDA level was significantly reduced after administrating of DPHC (*p* < 0.05). In addition, the SOD level of the DPHC (140 mg/kg) group was also increased but did not display a statistical difference. These results indicated that severe oxidative stress occurred in db/db mice, and DPHC exerted protective effect for liver tissues of db/db mice on oxidative stress damage by activating or inhibiting antioxidant enzyme activities.

#### Effect of DPHC on Liver Tissue Histopathological Changes and Hepatic Glycogen Content in db/db Mice

**FIGURE 5:**
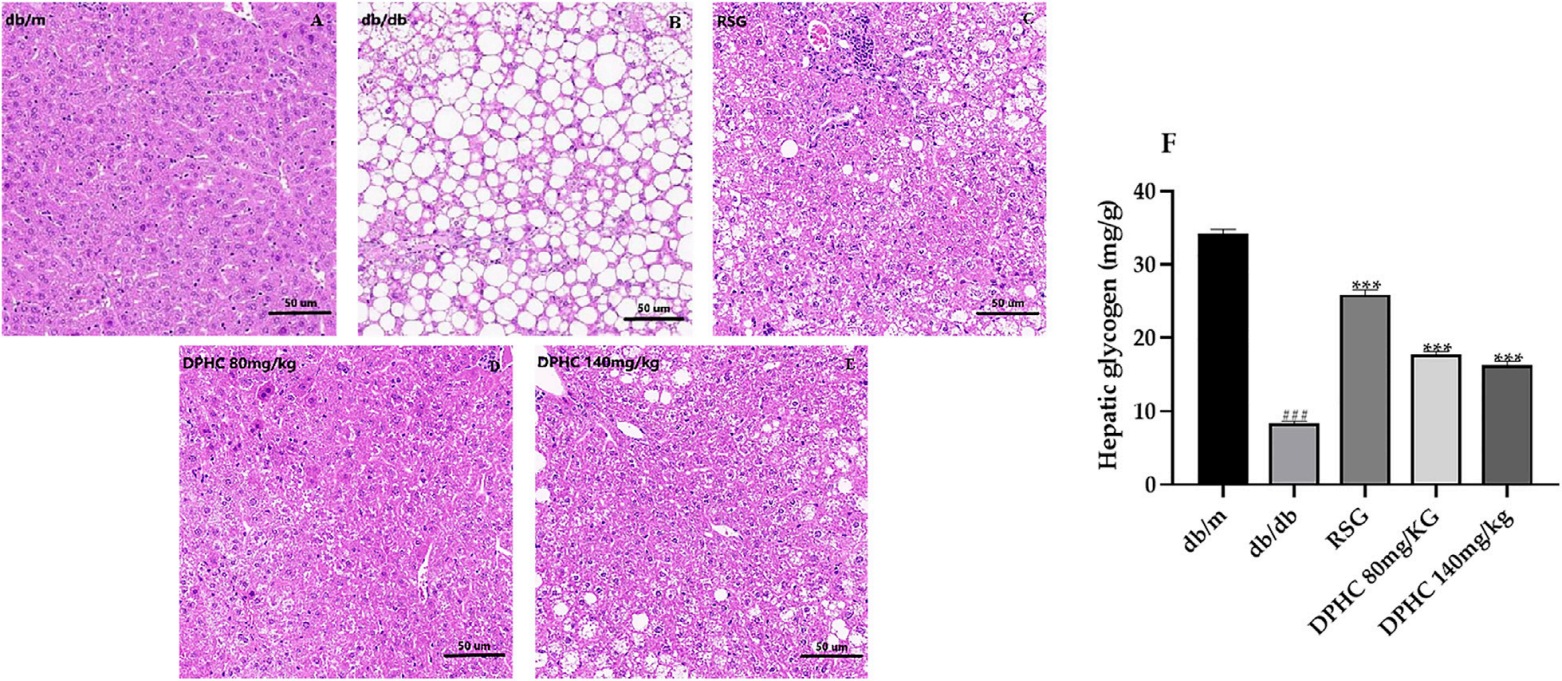
Effects of DPHC on liver tissue histopathological changes and hepatic glycogen contents in db/db mice. **(A–E)** Histopathological changes in the livers of mice. Magnification is 200×. **(F)** Liver glycogen levels in mice. All values are expressed as means ± SD, n = 9. ***p < 0.001 compared with the db/db group, and ^###^ *p* < 0.001 compared with the db/m group.

The effect of DPHC on the pathological changes of liver tissue is shown in Figures 5A–E. The liver lobules in the db/m group were clear, and the liver tissue structure was normal with a complete morphology. In contrast, liver cells in the db/db group were massively necrotic, and the cytoplasm was filled with circular lipid droplets of varying sizes. However, the numbers of vacuoles in DPHC groups were significantly reduced, which indicated that the accumulation of lipid droplets was less and the morphology of liver cells returned to a relatively normal state. The HE staining result revealed that liver tissue was protected from damage caused by IR in DPHC groups.

The liver glycogen levels in the liver tissues of mice are shown in Figure 5F. The liver glycogen content of the db/db group was significantly lower (*p* < 0.001) compared with the db/m group. After administration, the liver glycogen levels of DPHC and RSG groups were significantly increased (*p* < 0.001) compared with the db/db group. The above results showed that DPHC could reverse liver damage and promote glycogen synthesis in the livers of db/db mice.

#### Effects of DPHC on Ameliorating Oxidative Stress and IR *via* Nrf2/ARE Signal Pathway-Related Proteins in Liver Tissues of db/db Mice Through WB Detection

**FIGURE 6:**
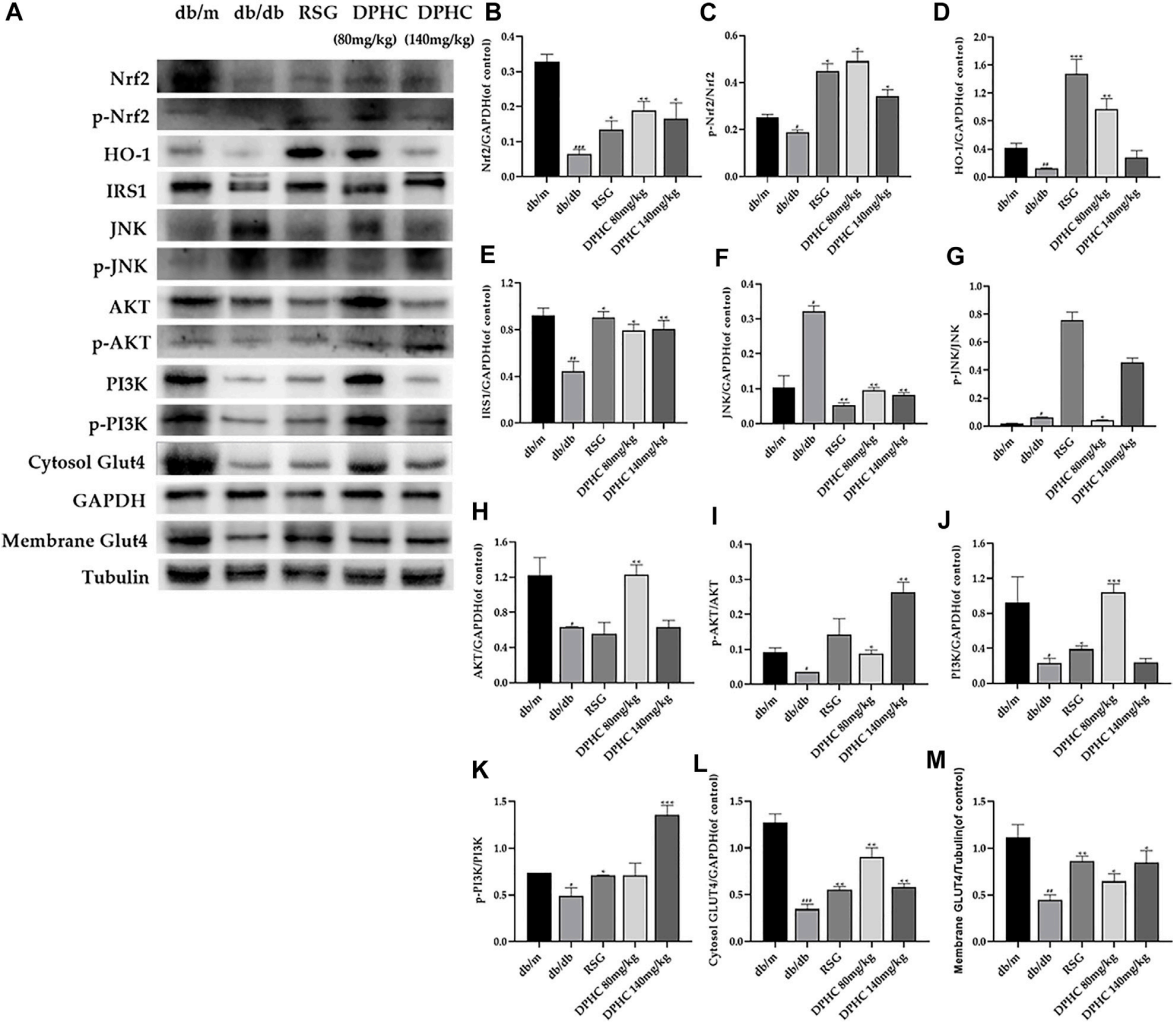
Effects of DPHC on ameliorating oxidative stress and IR *via* Nrf2/ARE signal pathway-related proteins in liver tissues of db/db mice. **(A)** The expression levels of Nrf2, *p*-Nrf2, HO-1, JNK, *p*-JNK, IRS1, AKT, *p*-AKT, PI3K, p-PI3K, cytosol GLUT4, and membrane GLUT4 in liver tissues of mice. **(B)** Nrf2. **(C)** The ratio of *p*-Nrf2/Nrf2. **(D)** HO-1. **(E)** IRS1. **(F)** JNK. **(G)** The ratio of *p*-JNK/JNK. **(H)** AKT. **(I)** The ratio of *p*-AKT/AKT. **(J)** PI3K. **(K)** The ratio of p-PI3K/PI3K. **(L)** Cytosol GLUT4. **(M)** Membrane GLUT4. All values are expressed as means ± SD, n = 3. *p < 0.05, **p < 0.01, ***p < 0.001 compared with the db/db group, and ^#^ *p* < 0.05, ^##^ *p* < 0.01, ^###^ *p* < 0.001 compared with the db/m group.

The results of all the target proteins were normalized to the GAPDH bond except membrane GLUT4, which was normalized to the tubulin bond. As shown in Figure 6A-M, the expression levels of Nrf2, *p*-Nrf2, HO-1, IRS1, AKT, *p*-AKT, PI3K, p-PI3K, cytosol GLUT4, and membrane GLUT4 in liver tissues of the db/db group were all significantly decreased (*p* < 0.05), and JNK and *p*-JNK were significantly increased (*p* < 0.05) compared with the db/m group. After DPHC treatment, the expression levels of Nrf2, *p*-Nrf2, HO-1, IRS1, AKT, *p*-AKT, PI3K, p-PI3K, cytosol GLUT4, and membrane GLUT4 were observably upregulated (*p* < 0.05), and the expression levels of JNK and *p*-JNK were observably downregulated (*p* < 0.01). These consequences indicated that the effect of improving oxidative stress and IR of DPHC in db/db mice was *via* activating the Nrf2/ARE signaling pathway.

#### Effects of DPHC on Improving Oxidative Stress and IR *via* Nrf2/ARE Signal Pathway-Related mRNA in Liver Tissues of db/db Mice Through qPCR Detection

**FIGURE 7:**
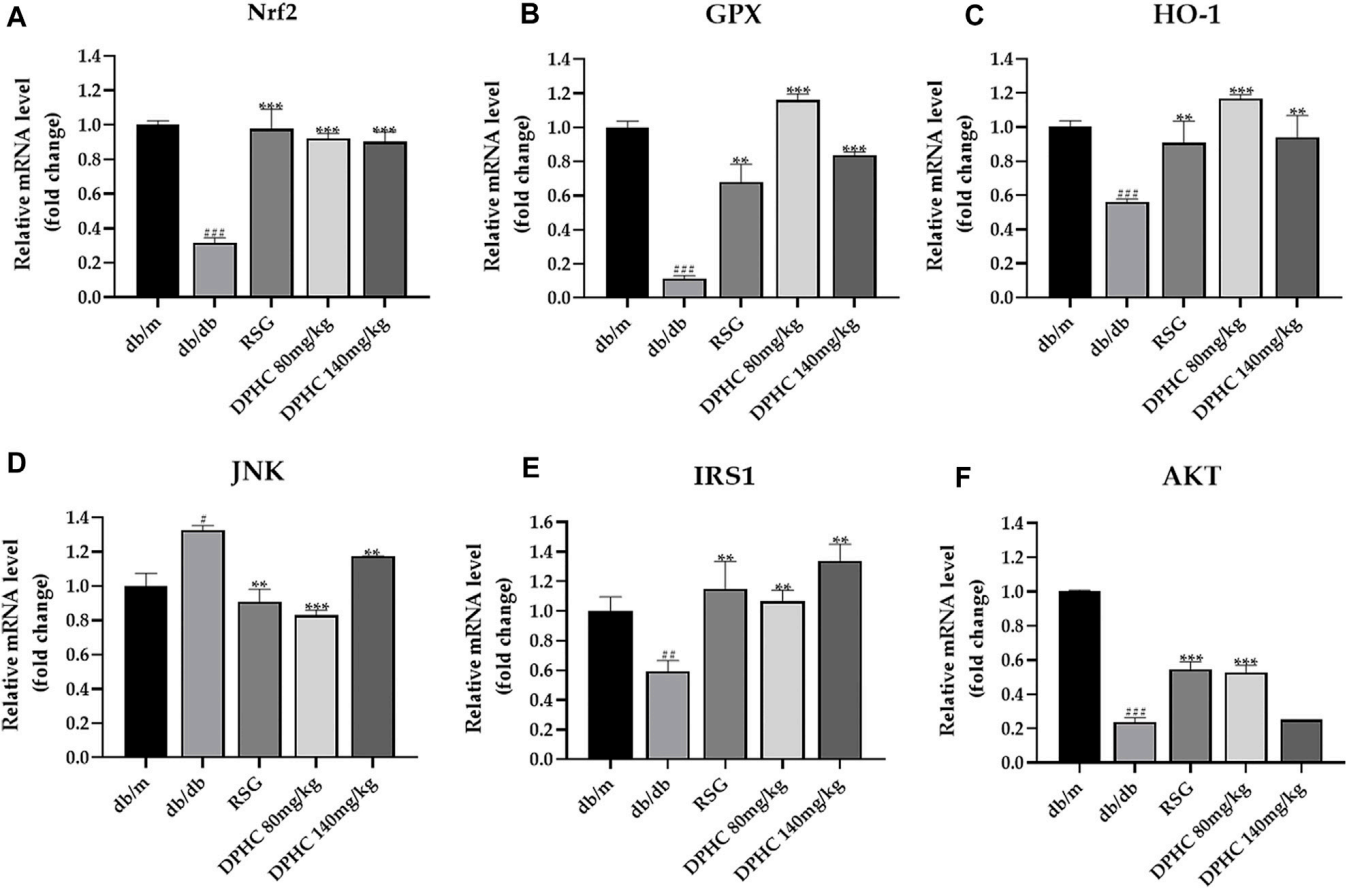
Effects of DPHC on improving oxidative stress and IR *via* Nrf2/ARE signal pathway-related mRNA in liver tissues of db/db mice. **(A)** *Nrf2*. **(B)** *GPX*. **(C)** *HO-1*. **(D)** *JNK*. **(E)** *IRS1*. **(F)** *AKT*. The results of different groups were normalized according to the results of db/m group. All values are expressed as means ± SD, n = 3. *p < 0.05, **p < 0.01, ***p < 0.001 compared with the db/db group, and ^##^ *p* < 0.01, ^###^ *p* < 0.001 compared with the db/m group.

*GAPDH* was used for the internal reference. The results of the db/m group were used to standardize the remaining groups. As depicted in Figures 7A–F, the expression levels of *Nrf2*, *GPX1*, *HO-1*, *IRS1*, and *AKT* in the db/db group were lower than those in the db/m group (*p* < 0.01), while *JNK* expression level was observably increased (*p* < 0.001). However, after dosing of DPHC and RSG, the expression levels of *Nrf2*, *GPX1*, *HO-1*, *IRS1*, and *AKT* were significantly increased (*p* < 0.05), and *JNK* level was significantly decreased (*p* < 0.05) in DPHC and RSG groups by comparison with the db/db group. Overall, these consequences indicated that DPHC could improve oxidative stress and IR of db/db mice mainly *via* activating Nrf2/ARE signaling pathway.

### *In Vitro* Study

#### Effect of Different Concentrations of DPHC on the Viability of HepG2 Cells

**FIGURE 8:**
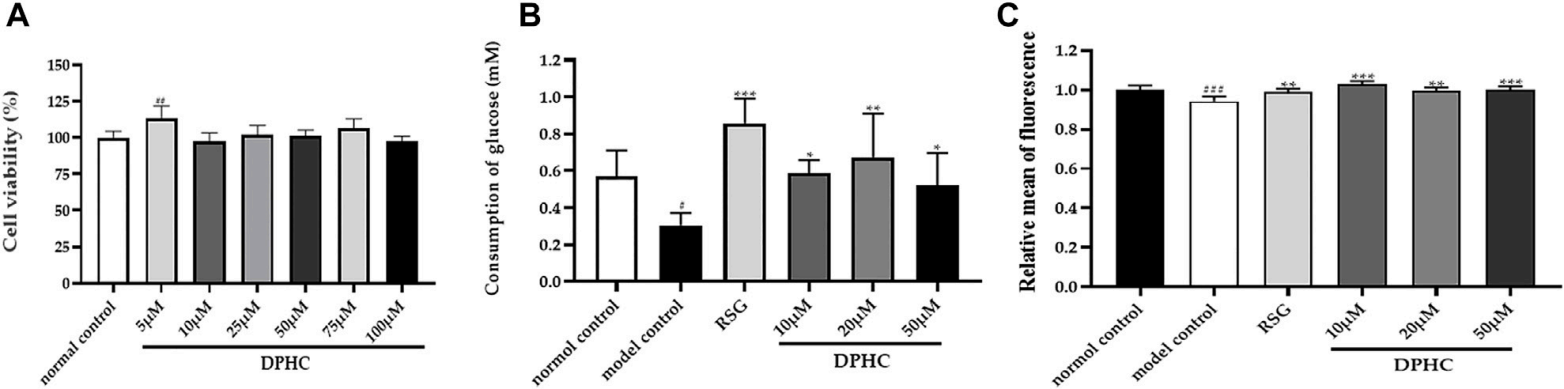
**(A)** Effect of different concentrations of DPHC on the viability of HepG2 cells for 24 h. **(B)** Glucose consumption. **(C)** Quantify fluorescence to show the results of glucose uptake. All values are expressed as means ± SD, n = 4. *p < 0.05, **p < 0.01, ***p < 0.001 compared with the model control group, and ^##^ *p* < 0.01, ^###^ *p* < 0.001 compared with the normal control group.

The CCK8 method was used to detect the effect of different concentrations of DPHC (5–100 µM) on the viability of HepG2 cells. As shown in Figure 8A, the cell viability in each group was not significantly reduced compared with the normal control group treated with various drug concentrations of DPHC for 24 h. Among them, DPHC had a certain promotion effect on cell growth at 5 µM (*p* < 0.01). Therefore, the result exhibited that DPHC at the concentration of 5–100 µM had no obvious toxic effect on the HepG2 cells. Hence, DPHC with doses of 10, 20, and 50 µM were selected to be used for the subsequent experiments.

#### Effects of DPHC on the Glucose Consumption and Glucose Uptake in IR-HepG2 Cells

In order to explore the effect of DPHC on improving IR induced by high glucose, the cellular glucose uptake and glucose consumption were detected in IR-HepG2 cells. In the experiment, glucose consumption and glucose intake of the model control group were significantly reduced (*p* < 0.05) compared with the normal control group as depicted in Figures 8B,C, which revealed that IR was successfully established in HepG2 cells. Then, the glucose consumption and glucose uptake of IR-HepG2 cells in DPHC (10, 20, and 50 µM) and RSG groups were significantly increased (*p* < 0.05). Hence, the results indicated that DPHC could improve the glucose metabolism and enhance insulin sensitivity of IR-HepG2 cells induced by high glucose.

#### Effect of DPHC on Oxidative Stress in IR-HepG2 Cells

**FIGURE 9:**
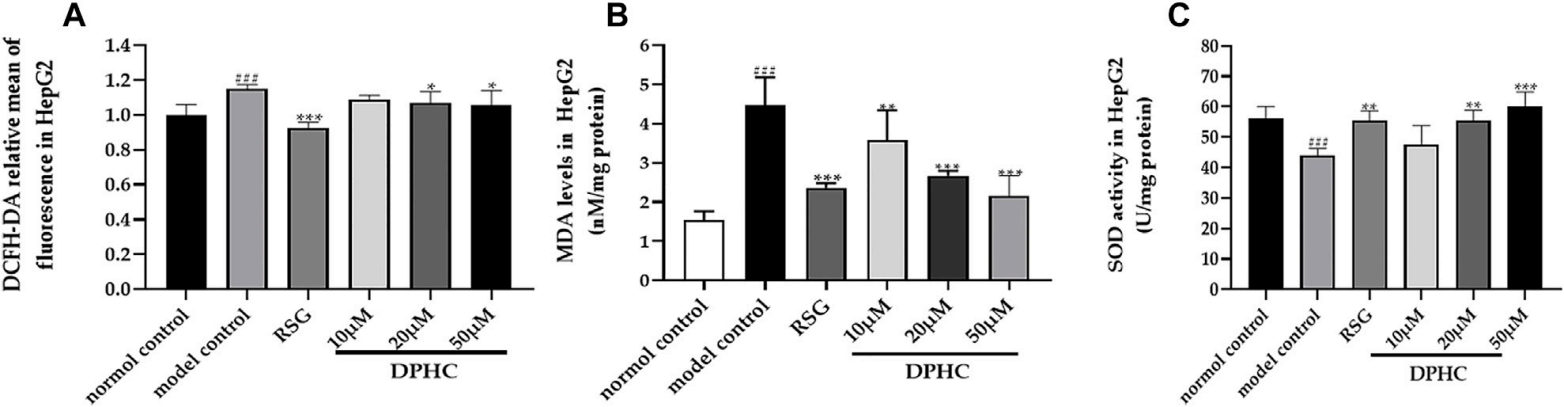
Effects of DPHC on intracellular ROS, MDA, and SOD levels in IR-HepG2 cells. **(A)** ROS. **(B)** MDA. **(C)** SOD. All values are expressed as means ± SD, n = 4. *p < 0.05, **p < 0.01, ***p < 0.001 compared with the model control group, and ^###^ *p* < 0.001 compared with the normal control group.

Oxidative stress was considered to be one of the main causes of IR. In order to investigate the effect of DPHC on improving oxidative stress, the levels of ROS, MDA, and SOD in IR-HepG2 cells were measured. As shown in Figures 9A–C, the SOD level of the model control group was significantly reduced (*p* < 0.001), and ROS and MDA levels were significantly increased (*p* < 0.001) compared with the normal control group. However, the activities of SOD in DPHC (20 and 50 µM) and RSG groups were significantly increased, and intracellular ROS level was significantly reduced (*p* < 0.05) compared with the model control group. Moreover, MDA contents in DPHC (10, 20, and 50 µM) and RSG groups were significantly reduced (*p* < 0.01) compared with the model control group. The above-mentioned results indicated that oxidative stress was induced in IR-HepG2 cells on account of high doses of glucose, and DPHC could quench the intracellular ROS and regulate related antioxidant enzymes to protect cells from oxidative stress.

#### Effects of DPHC on Ameliorating Oxidative Stress and IR *via* Nrf2/ARE Signal Pathway-Related Proteins in IR-HepG2 Cells Through WB Detection

**FIGURE 10:**
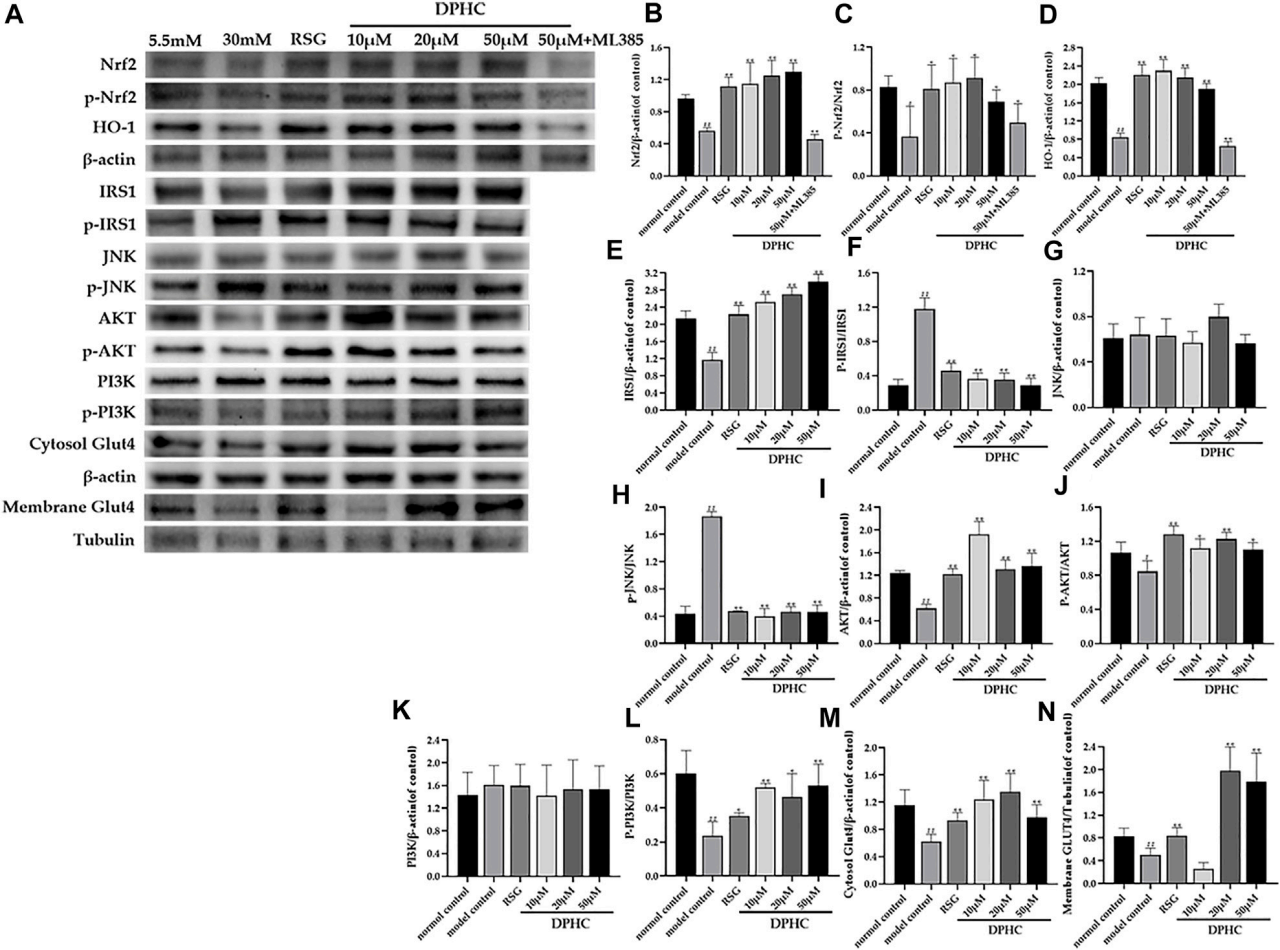
Effects of DPHC on ameliorating oxidative stress and IR via Nrf2/ARE signal pathway-related proteins in IR-HepG2 cells. **(A)** The expression levels of Nrf2, *p*-Nrf2, HO-1, JNK, *p*-JNK, IRS1, AKT, *p*-AKT, PI3K, p-PI3K, cytosol GLUT4, and membrane GLUT4 in liver tissues of mice. **(B)** Nrf2. **(C)** The ratio of *p*-Nrf2/Nrf2. **(D)** HO-1. **(E)** IRS1. **(F)** The ratio of p-IRS1/IRS1. **(G)** JNK. **(H)** The ratio of *p*-JNK/JNK. **(I)** AKT. **(J)** The ratio of *p*-AKT/AKT. **(K)** PI3K. **(L)** The ratio of p-PI3K/PI3K. **(M)** Cytosol GLUT4. **(N)** Membrane GLUT4. All values are expressed as means ± SD, n = 3. *p < 0.05, **p < 0.01 compared with the model control group, and ^#^ *p* < 0.05, ^##^ *p* < 0.01 compared with the normal control group.

In the studies, the membrane GLUT4 was normalized to the tubulin bond, and other proteins were normalized to the β-actin bond. As shown in Figure 10A-N, the protein levels of Nrf2, *p*-Nrf2, HO-1, IRS1, AKT, *p*-AKT, p-PI3K, cytosol GLUT4, and membrane GLUT4 in IR-HepG2 cells of the model control group were all significantly decreased (*p* < 0.05), and the protein levels of p-IRS1on Ser307 and *p*-JNK were significantly increased (*p* < 0.05) compared with the normal control group. However, the protein levels of Nrf2, *p*-Nrf2, HO-1, IRS1, AKT, *p*-AKT, p-PI3K, cytosol GLUT4, and membrane GLUT4 were significantly upregulated (*p* < 0.05), and the protein levels of p-IRS1 on Ser 307 and *p*-JNK protein were significantly downregulated (*p* < 0.01) after DPHC treatment. Moreover, the expression levels of Nrf2 and HO-1 in the DPHC (50 µm) with Nrf2 inhibitor (ML385) group were not significantly different from those in the model control group (*p* > 0.05). Finally, these results were generally consistent with the *in vivo* experiments, which indicated that DPHC could improve oxidative stress and IR *in vitro* by activating the Nrf2/ARE signaling pathway.

#### Effects of DPHC on Ameliorating Oxidative Stress and IR *via* Nrf2/ARE Signal Pathway-Related mRNA in IR-HepG2 Cells Through qPCR Detection

**FIGURE 11:**
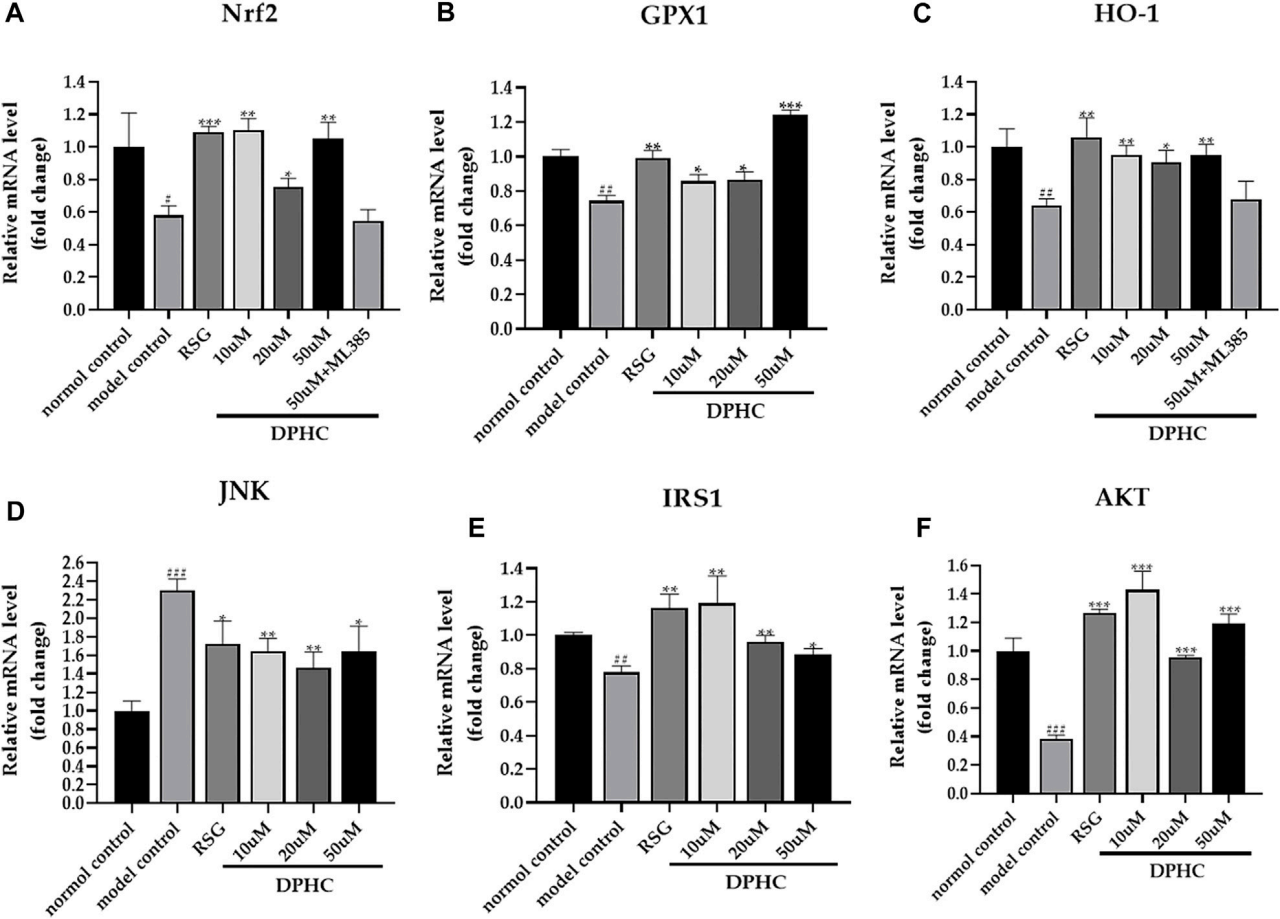
Effects of DPHC on ameliorating oxidative stress and IR *via* Nrf2/ARE signal pathway-related mRNA in IR-HepG2 cells. **(A)** *Nrf2*. **(B)** *GPX*. **(C)** *HO-1*. **(D)** *JNK*. **(E)** *IRS1*. **(F)** *AKT*. The results of different groups were normalized according to the results of the normal control group. All values are expressed as means ± SD, n = 3. *p < 0.05, **p < 0.01, ***p < 0.001 compared with the model control group, and ^##^ *p* < 0.01, ^###^ *p* < 0.001 compared with the normal control group.

The internal reference was *GAPDH*. The result of the normal control group was used to standardize the remaining groups. As shown in Figures 11A–F, the mRNA expression levels of *Nrf2*, *GPX1*, *HO-1*, *IRS1*, and *AKT* in the IR-HepG2 cells of the model control group were all significantly decreased (*p* < 0.05), while *JNK* expression level was significantly increased (*p* < 0.001) compared with the normal control group. However, the levels of *Nrf2*, *GPX1*, *HO-1*, *IRS1*, and *AKT* in DPHC (10, 20, and 50 µM) and RSG groups were significantly increased (*p* < 0.05), and *JNK* level was significantly decreased (*p* < 0.05) after administration of DPHC. Moreover, the expression levels of *Nrf2* and *HO-1* in DPHC (50 µm) with Nrf2 inhibitor (ML385) group were not significantly disparate from those in the model control group (*p* > 0.05). These results also continued to support that the improvement effects on oxidative stress and IR of DPHC were based on activating Nrf2/ARE signaling pathway.

## Discussion

T2DM accounts for more than 90% of diabetes cases and has become a complex and highly morbid condition worldwide with hyperglycemia and hyperlipidemia (Whiting et al., 2011; Ma and Chan, 2013; Xu et al., 2013; Schreiber et al., 2015). The generation of oxidative stress has been proven to be one of the causes of long-term high blood glucose level and a series of diabetic complications (Evans et al., 2002) and was considered to be a key factor to lead to IR and induce T2DM (Yang et al., 2014). The db/db mice are in a hyperglycemic state due to the lack of leptin receptor genes with spontaneous symptoms of T2DM, which is similar to clinical T2DM cases (Guilbaud et al., 2019). Our previous preliminary pre-experiment result proved that DPHC has a hypoglycemic effect on db/db mice in the dose range of 70–160 mg/kg. Therefore, the doses of 80 and 140 mg/kg were applied for this study and intervened for 8 weeks. Experimental results showed that FBG level in db/db mice was significantly increased with the occurrence of IR, and this result was consistent with previous report (Loffler et al., 2006). Nevertheless, it is worth noting that DPHC significantly reduced FBG level in db/db mice, which exhibited a similar effect to RSG. Furthermore, the glucose tolerance and insulin sensitivity of db/db mice were significantly improved after treatment with DPHC, and the serum insulin of db/db mice was significantly reduced. HOMA-IR and ISI were analyzed by homeostasis assessment methods, which represented the efficiency of insulin utilization in the body and the health of pancreatic β-cells (Gao et al., 2017), respectively. Our experimental results displayed that DPHC could ameliorate glucose tolerance and IR, thereby accelerated glucose metabolism and reduced FBG level in db/db mice. Furthermore, the effectiveness of DPHC was also validated *in vitro*. HepG2 cells are common hepatocyte models for the investigation of the hypoglycemic effects of drugs (Chen et al., 2018). Our consequences proved that DPHC observably promoted glucose consumption and glucose uptake in IR-HepG2 cells with no cytotoxicity, further proving that DPHC could improve glucose metabolism.

Furthermore, oxidative stress status was assessed after DPHC administration *in vivo* and *in vitro*. High glucose in the body will cause excessive accumulation of ROS and eventually lead to oxidative stress and IR (Poitout and Robertson, 2008). Therefore, directly detecting the production of ROS in cells is an important means to evaluate cell oxidative damage. In the study, intracellular ROS level in the model control group was significantly increased compared with the normal control group, while the increase in ROS was significant reversed after DPHC treatment in IR-HepG2 cells. Antioxidant ability was also evaluated by detecting the changes in antioxidant enzyme activities. MDA reflects the degree of lipid peroxidation in the body and succinctly reflect the degree of cell damage (Zhang et al., 2020). SOD and CAT are the main antioxidant enzymes that protect cells from harmful ROS. GSH-Px plays an important role in protecting cell membranes from lipid peroxidative damage (Jiang and Yang, 2003). Therefore, we detected the levels of SOD, CAT, and GSH-Px in the liver tissues of db/db mice and SOD and MDA in IR-HepG2 cells to reflect the oxidative stress *in vivo* and *in vitro* environments. In *in vivo* experiments, the levels of SOD, CAT, and GSH-Px in the db/db group were significantly reduced, and the MDA level was significantly increased compared with the db/m group, which proved that severe IR was produced in the db/db mice. However, the SOD and CAT levels in the DPHC groups were significantly increased, and the MDA levels of DPHC groups were significantly reduced after treatment. Furthermore, the results of *in vitro* experiments showed that the SOD level of IR-HepG2 cells induced by high glucose in the model control group was significantly reduced, and the MDA level was significantly increased compared with the normal control group. Nevertheless, the SOD activity of IR-HepG2 cells was significantly increased, and the intracellular MDA level was significantly declined after DPHC treatment. These results exhibited that severe oxidative stress was induced in db/db mice and IR-HepG2 cells, while DPHC treatment could protect the body from oxidative stress damage by activating or inhibiting the activity of corresponding antioxidant enzymes.

The liver is one of the important target tissues for insulin to play a role in the development of T2DM (Cai et al., 2018). Liver glycogen is maintained at normal level in the body by supplying glucose to the blood or synthesizing glycogen in the liver, which is essential for blood glucose homeostasis (Adeva-Andany et al., 2016). Whereupon, we detected the liver glycogen content and performed H&E staining on the liver of db/db mice. The results revealed that the liver glycogen content of db/db mice was significantly reduced due to the reduced utilization of glucose by hepatocytes. However, glycogen synthesis was increased, which led to the addition of glycogen content after DPHC intervention. H&E staining results exhibited that the liver lobules in the db/m group were clear, and the liver tissue structure was normal with a complete morphology. In contrast, liver cells were massively necrotic, and the cytoplasm was filled with circular lipid droplets of varying sizes in the db/db group. Furthermore, the liver tissue was damaged, and the morphology and structure were abnormal. Nevertheless, the numbers of vacuoles in DPHC groups were significantly reduced, which indicated that the accumulation of lipid droplets was less and the morphology of liver cells returned to a relatively normal state.

Finally, the molecular mechanism of DPHC on improving oxidative stress and IR was investigated, and the related gene and protein expressions were detected by qPCR and WB in the liver tissues of db/db mice and IR-HepG2 cells. Nrf2 binding to ARE could activate a series of antioxidant reaction to resist oxidative stress *via* initiating SOD, CAT, glutathione, NADPH (Bhakkiyalakshmi et al., 2015), and HO-1 (Zhu et al., 2020) while directly reducing excess ROS (Aleksunes et al., 2010) to protect and restore cell homeostasis under various stimuli (Uruno et al., 2016), while the suppression of Nrf2 in the body increases blood glucose level, aggravates glucose intolerance, and inhibits insulin signaling (Uruno et al., 2015). In our investigation, the protein and gene expression levels of Nrf2 and HO-1 were significantly increased after DPHC treatment in the liver tissues of db/db mice and IR-HepG2 cells. However, DPHC was no longer effective after Nrf2 protein, and gene expression were inhibited in DPHC (50 µm) with Nrf2 inhibitor (ML385) group, and it was not significantly different from the model control group, which further proved that the improving effect on oxidative stress and IR of DPHC was through activating Nrf2/ARE signaling pathway. In addition, the activation of JNK, a member of the mitogen-activated protein kinase family, could increase ROS and play a vital in the body’s resistance to oxidative stress and inhibit insulin signal transduction and glucose uptake (EL-Beltagi and Mohamed, 2013). Thus, the influence of DPHC on JNK level was further evaluated. As shown in consequences, the levels of JNK and *p*-JNK were significantly elevated in db/db mice and IR-HepG2 cells, but DPHC reversed the increases in JNK and *p*-JNK. Besides, IRS1 is also a key target of insulin receptor tyrosine kinase and gene locus necessary for controlling metabolism hormones (Scheen, 2014). JNK and high glucose could stimulate phosphorylation of IRS1 (p-IRS1) on Ser307, which reduces IRS1 function and causes IR (Gual et al., 2005). Furthermore, phosphorylation of IRS1 activates PI3K and regulates the downstream factor AKT and thus promotes glucose uptake (Matsumoto et al., 2002; Zhang et al., 2018). The PI3K/AKT signaling pathway is a classic insulin signaling pathway that can modulate glucose transport by activating insulin (Sadi et al., 2018). Based on the above analysis, the expression levels of related proteins and genes in the IRS1/PI3K/AKT signaling pathway were explored. The results showed that the protein expression levels of IRS1, AKT, *p*-AKT, PI3K, and p-PI3K in the liver tissues of db/db mice and IR-HepG2 cells in DPHC groups were significantly increased, and the protein expression level of p-IRS1 on Ser 307 was markedly reduced. At the same time, the expressions of related genes showed similar effects. In addition, GLUT4, a member of the GLUTs family, maintains glucose balance by moving to the plasma membrane and promoting the uptake and transport of glucose into the cells on the membrane when regulated by IRS-1/PI3K/AKT signaling pathway (Huang and Czech, 2007; Guo et al., 2019). Our results showed that the GLUT4 protein expression level were significantly increased with high translocation rate from cytosol to membrane in the liver tissues of db/db mice and IR-HepG2 cells to exert to balance blood glucose.

## Conclusion

Overall, our investigation proved that DPHC from *Alpinia officinarum* could regulate blood glucose level and glucose tolerance in db/db mice and simultaneously improve oxidative stress to reduce tissue damage. *In vitro*, DPHC also had the same effects on improving glucose metabolism, IR, and oxidative stress in IR-HepG2 cells induced by high glucose. Furthermore, *in vivo* and *in vitro* experiments demonstrated that DPHC could activate Nrf2, *p*-Nrf2, HO-1, IRS1, AKT, *p*-AKT, p-PI3K, cytosol GLUT4, and membrane GLUT4 and downregulate p-IRS1 on Ser 307 and *p*-JNK levels. Therefore, DPHC has the potential to ameliorate oxidative stress and IR to treat T2DM *via* activating the Nrf2/ARE signaling pathway.

## Data Availability

The original data supporting the conclusions of this article will be provided by the author to any qualified researcher without reservation, and some of the data will be included in the supplementary materials.
